# Bis(benzo-15-crown-5-κ^5^ *O*)barium tetra­kis­(iso­thio­cyanato-κ*N*)cobaltate(II)

**DOI:** 10.1107/S2414314621000249

**Published:** 2021-01-12

**Authors:** V. Ravisankar, V. Ramesh, M. Krishnamohan, B. Gunasekaran, T. C. Sabari Girisun

**Affiliations:** a Department of Physics & Nano Technology, SRM Institute of Science and Technology, SRM Nagar, Kattankulathur, Kancheepuram Dist, Chennai-603 203 Tamil Nadu, India; b Nanophotonics Laboratory, School of Physics, Bharathidasan University, Tiruchirappalli, Tamil Nadu, India, 620 024; Goethe-Universität Frankfurt, Germany

**Keywords:** dibenzo 15-Crown-5 ether, cobalt thio­cyanide, catalysis and electrolyte, crystal structure

## Abstract

The title compound, [Ba(C_14_H_20_O_5_)_2_][Co(NCS)_4_], is formed by discrete anions and cations. The molecular packing is controlled by weak C—H⋯π inter­actions.

## Abstract 2

The title compound is formed by discrete anions and cations. The crystal packing is controlled by weak inter­molecular C—H⋯π inter­actions.

## Graphical abstract

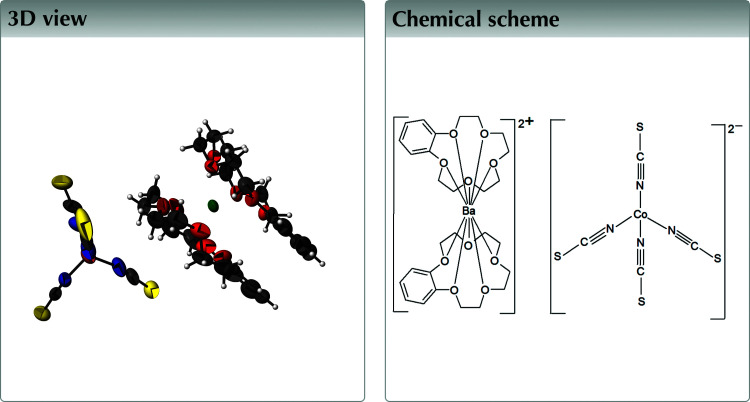

## Structure description

15-Crown-5 ether derivatives are used in the electronics industry, in phase-transfer catalysis (Alasundkar *et al.*, 2011 ▸), toxic metal sequestration and as battery electrolytes (Ligon *et al.*, 2004 ▸).

**Figure 1:**
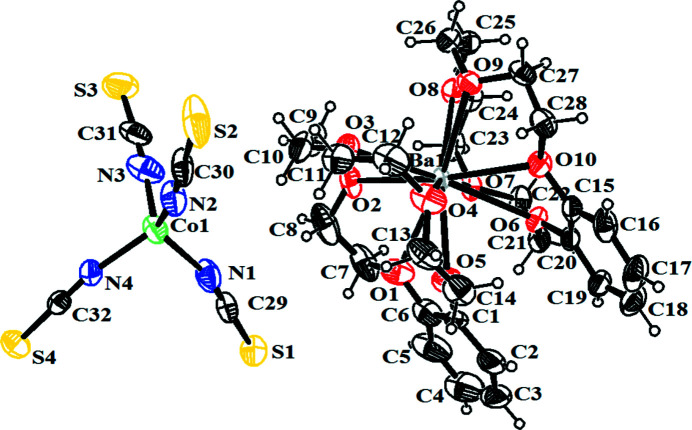
The mol­ecular structure of the title compound with atom labels and 30% probability displacement ellipsoids for non-H atoms.

The geometric parameters of the title compound (Fig. 1 ▸) agree well with those of reported similar structures (Cao *et al.*, 2010 ▸; Vafaee *et al.*, 2012 ▸; Ramesh *et al.*, 2019 ▸). The Co^II^ atom is surrounded by four N atoms and the Ba^II^ ion is coordinated by ten O atoms from two dibenzo-15-crown ligands in a sandwich-like configuration. Weak C—H⋯π inter­actions [C8⋯*Cg*1^i^ = 3.760 (7) Å, H8*A*⋯*Cg*1^i^ = 2.82 Å; C13⋯*Cg*2^ii^ = 3.546 (6) Å, H13*A*⋯*Cg*2^ii^ = 2.74 Å, *Cg*1 and *Cg*2 are the centroids of the C15–C20 and C1–C6 rings, respectively; symmetry codes: (i) 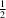 − *x*, 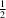 + *y*, 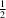 − *z*; (ii) 1 − *x*, 1 − *y*, 1 − *z*] stabilize the crystal.

## Synthesis and crystallization

Cobalt(II) chloride (0.25 mmol, 59.49 mg) and ammonium thio­cyanate (1 mmol, 76.12 mg) were dissolved in deionized (DI) water. An aqueous solution (5 ml) of barium(II) chloride (0.25 mmol, 52.07 mg) was added dropwise and the mixture was stirred for 3 h. Then a 1,2-di­chloro­ethane solution (10 ml) of benzo-15-crown-5 (0.5 mmol, 134.15 mg) was added dropwise. Finally, the solution was filtered using Whatman filter paper and the clear solution was held at room temperature for about 15 days when transparent blue crystals were obtained.

## Refinement

Crystal data, data collection and structure refinement details are summarized in Table 1 ▸.

## Supplementary Material

Crystal structure: contains datablock(s) I. DOI: 10.1107/S2414314621000249/bt4105sup1.cif

Structure factors: contains datablock(s) I. DOI: 10.1107/S2414314621000249/bt4105Isup2.hkl

CCDC reference: 1966145

Additional supporting information:  crystallographic information; 3D view; checkCIF report

## Figures and Tables

**Table 1:** Experimental details

Crystal data	
Chemical formula	[Ba(C_14_H_20_O_5_)_2_][Co(NCS)_4_]
*M*_r_	965.19
Crystal system, space group	Monoclinic, *P*2_1_/*n*
Temperature (K)	296
*a*, *b*, *c* (Å)	12.0501 (19), 20.208 (3), 17.367 (3)
β (°)	101.052 (4)
*V* (Å^3^)	4150.6 (11)
*Z*	4
Radiation type	Mo *K*α
μ (mm^−1^)	1.60
Crystal size (mm)	0.20 × 0.20 × 0.15

Data collection	
Diffractometer	Bruker APEXII CCD
Absorption correction	Multi-scan (*SADABS*; Bruker, 2008 ▸)
*T*_min_, *T*_max_	0.734, 0.787
No. of measured, independent and observed [*I* > 2σ(*I*)] reflections	62475, 8674, 6117
*R*_int_	0.065
(sin θ/λ)_max_ (Å^−1^)	0.630

Refinement	
*R*[*F* ^2^ > 2σ(*F* ^2^)], *wR*(*F* ^2^), *S*	0.039, 0.105, 1.06
No. of reflections	8674
No. of parameters	469
No. of restraints	24
H-atom treatment	H-atom parameters constrained
Δρ_max_, Δρ_min_ (e Å^−3^)	0.62, −0.54

Computer programs: *APEX2* and *SAINT* (Bruker, 2008 ▸), *SHELXT* (Sheldrick, 2015*a* ▸), *SHELXL2018/3* (Sheldrick, 2015*b* ▸), *XP* in *SHELXTL* (Sheldrick, 2008 ▸) and *PLATON* (Spek, 2020 ▸).

## full crystallographic data

### Crystal data

**Table:**

[Ba(C_14_H_20_O_5_)_2_][Co(NCS)_4_]	*F*(000) = 1948
*M**_r_* = 965.19	*D*_x_ = 1.545 Mg m^−^^3^
Monoclinic, *P*2_1_/*n*	Mo *K*α radiation, λ = 0.71073 Å
*a* = 12.0501 (19) Å	Cell parameters from 8674 reflections
*b* = 20.208 (3) Å	θ = 1.6–26.6°
*c* = 17.367 (3) Å	µ = 1.60 mm^−^^1^
β = 101.052 (4)°	*T* = 296 K
*V* = 4150.6 (11) Å^3^	Block, blue
*Z* = 4	0.20 × 0.20 × 0.15 mm

### Data collection

**Table:**

Bruker APEXII CCD diffractometer	6117 reflections with *I* > 2σ(*I*)
ω and φ scans	*R*_int_ = 0.065
Absorption correction: multi-scan (SADABS; Bruker, 2008)	θ_max_ = 26.6°, θ_min_ = 1.6°
*T*_min_ = 0.734, *T*_max_ = 0.787	*h* = −15→15
62475 measured reflections	*k* = −25→25
8674 independent reflections	*l* = −21→21

### Refinement

**Table:**

Refinement on *F*^2^	Primary atom site location: structure-invariant direct methods
Least-squares matrix: full	Hydrogen site location: inferred from neighbouring sites
*R*[*F*^2^ > 2σ(*F*^2^)] = 0.039	H-atom parameters constrained
*wR*(*F*^2^) = 0.105	*w* = 1/[σ^2^(*F*_o_^2^) + (0.0375*P*)^2^ + 5.4623*P*] where *P* = (*F*_o_^2^ + 2*F*_c_^2^)/3
*S* = 1.06	(Δ/σ)_max_ = 0.001
8674 reflections	Δρ_max_ = 0.62 e Å^−^^3^
469 parameters	Δρ_min_ = −0.54 e Å^−^^3^
24 restraints	

### Special details

**Table:**

Geometry. All esds (except the esd in the dihedral angle between two l.s. planes) are estimated using the full covariance matrix. The cell esds are taken into account individually in the estimation of esds in distances, angles and torsion angles; correlations between esds in cell parameters are only used when they are defined by crystal symmetry. An approximate (isotropic) treatment of cell esds is used for estimating esds involving l.s. planes.
Refinement. The H atoms were positioned geometrically and refined using a riding model with C—H = 0.97 Å and *U*_iso_(H) = 1.2Ueq(C) for CH_2_ and C—H = 0.93 Å and *U*_iso_(H) = 1.2Ueq(C) for CH.

### Fractional atomic coordinates and isotropic or equivalent isotropic displacement parameters (Å^2^)

**Table:**

	*x*	*y*	*z*	*U*_iso_*/*U*_eq_	
C1	0.3225 (6)	0.4533 (3)	0.4357 (3)	0.0824 (19)	
C2	0.2934 (7)	0.4214 (4)	0.5002 (4)	0.129 (3)	
H2	0.333921	0.385141	0.523499	0.155*	
C3	0.1987 (9)	0.4469 (5)	0.5287 (5)	0.130 (4)	
H3	0.173072	0.426837	0.570131	0.156*	
C4	0.1461 (9)	0.5031 (7)	0.4921 (7)	0.173 (6)	
H4	0.086649	0.521102	0.512321	0.207*	
C5	0.1753 (6)	0.5334 (5)	0.4292 (5)	0.135 (3)	
H5	0.136534	0.570588	0.406899	0.162*	
C6	0.2646 (5)	0.5074 (4)	0.3992 (4)	0.0873 (19)	
C7	0.2550 (5)	0.5918 (3)	0.2971 (5)	0.110 (2)	
H7A	0.243978	0.625561	0.334624	0.132*	
H7B	0.182835	0.582531	0.263286	0.132*	
C8	0.3400 (6)	0.6148 (3)	0.2491 (5)	0.115 (2)	
H8A	0.315582	0.656205	0.222892	0.138*	
H8B	0.413232	0.621721	0.282665	0.138*	
C9	0.4405 (7)	0.5831 (3)	0.1506 (4)	0.111 (2)	
H9A	0.428222	0.557825	0.102204	0.133*	
H9B	0.436920	0.629714	0.136848	0.133*	
C10	0.5539 (7)	0.5679 (4)	0.1962 (4)	0.115 (2)	
H10A	0.610700	0.575690	0.164558	0.138*	
H10B	0.570379	0.596120	0.242193	0.138*	
C11	0.6627 (4)	0.4851 (4)	0.2663 (4)	0.097 (2)	
H11A	0.677081	0.512469	0.313037	0.117*	
H11B	0.722755	0.492800	0.237311	0.117*	
C12	0.6620 (5)	0.4151 (4)	0.2888 (5)	0.107 (2)	
H12A	0.655492	0.388075	0.241988	0.128*	
H12B	0.733691	0.404552	0.322601	0.128*	
C13	0.5984 (5)	0.4072 (3)	0.4104 (3)	0.0935 (18)	
H13A	0.625692	0.451895	0.422542	0.112*	
H13B	0.657747	0.376773	0.433564	0.112*	
C14	0.5007 (6)	0.3960 (3)	0.4438 (4)	0.0953 (18)	
H14A	0.481154	0.349386	0.441194	0.114*	
H14B	0.514924	0.409652	0.498369	0.114*	
C15	0.3350 (4)	0.2725 (2)	0.3200 (3)	0.0579 (12)	
C16	0.3861 (5)	0.2228 (3)	0.3700 (4)	0.0824 (17)	
H16	0.451338	0.201879	0.361345	0.099*	
C17	0.3369 (7)	0.2053 (3)	0.4331 (4)	0.102 (2)	
H17	0.370996	0.173143	0.468060	0.123*	
C18	0.2403 (7)	0.2342 (4)	0.4446 (4)	0.101 (2)	
H18	0.207014	0.220258	0.485946	0.122*	
C19	0.1900 (5)	0.2845 (3)	0.3955 (3)	0.0780 (16)	
H19	0.124790	0.305232	0.404520	0.094*	
C20	0.2381 (4)	0.3031 (2)	0.3334 (3)	0.0546 (11)	
C21	0.0868 (4)	0.3802 (2)	0.2793 (3)	0.0643 (12)	
H21A	0.089826	0.414993	0.318184	0.077*	
H21B	0.035533	0.346017	0.290289	0.077*	
C22	0.0484 (4)	0.4072 (3)	0.1993 (3)	0.0686 (13)	
H22A	0.041586	0.371700	0.161025	0.082*	
H22B	−0.025130	0.427863	0.195347	0.082*	
C23	0.1058 (4)	0.4772 (3)	0.1035 (3)	0.0768 (15)	
H23A	0.141414	0.519944	0.100539	0.092*	
H23B	0.024920	0.482872	0.086384	0.092*	
C24	0.1482 (4)	0.4301 (3)	0.0501 (3)	0.0731 (14)	
H24A	0.112403	0.387229	0.051869	0.088*	
H24B	0.130626	0.446292	−0.003450	0.088*	
C25	0.3166 (5)	0.3807 (3)	0.0265 (3)	0.0721 (14)	
H25A	0.305917	0.398026	−0.026431	0.087*	
H25B	0.281135	0.337440	0.024656	0.087*	
C26	0.4403 (4)	0.3751 (3)	0.0607 (3)	0.0729 (14)	
H26A	0.475369	0.345295	0.028689	0.088*	
H26B	0.475374	0.418199	0.059655	0.088*	
C27	0.4552 (4)	0.2808 (2)	0.1461 (3)	0.0676 (13)	
H27A	0.516820	0.260792	0.125836	0.081*	
H27B	0.384545	0.264631	0.115474	0.081*	
C28	0.4632 (4)	0.2623 (2)	0.2285 (3)	0.0691 (13)	
H28A	0.454499	0.214804	0.233253	0.083*	
H28B	0.536094	0.275077	0.258992	0.083*	
C29	0.4800 (5)	0.7298 (2)	0.3803 (4)	0.0736 (15)	
C30	0.8498 (4)	0.6363 (3)	0.2821 (5)	0.090 (2)	
C31	0.6233 (5)	0.7841 (4)	0.1279 (4)	0.096 (2)	
C32	0.7502 (4)	0.8760 (2)	0.3929 (3)	0.0555 (11)	
Ba1	0.36293 (2)	0.43398 (2)	0.23540 (2)	0.04227 (9)	
Co1	0.68526 (6)	0.74686 (4)	0.30213 (5)	0.0757 (2)	
N1	0.5546 (4)	0.7252 (3)	0.3488 (4)	0.0995 (18)	
N2	0.7929 (4)	0.6751 (2)	0.3021 (3)	0.0913 (16)	
N3	0.6391 (5)	0.7691 (3)	0.1925 (4)	0.112 (2)	
N4	0.7438 (4)	0.8265 (2)	0.3593 (3)	0.0687 (11)	
O1	0.3025 (3)	0.5312 (2)	0.3380 (2)	0.0879 (12)	
O2	0.3470 (4)	0.56720 (17)	0.1957 (3)	0.0924 (14)	
O3	0.5554 (3)	0.5029 (2)	0.2185 (2)	0.0908 (12)	
O4	0.5746 (3)	0.3984 (2)	0.3277 (2)	0.0893 (12)	
O5	0.4133 (3)	0.43300 (18)	0.4009 (2)	0.0773 (10)	
O6	0.1977 (2)	0.35324 (14)	0.28096 (18)	0.0567 (8)	
O7	0.1287 (2)	0.45466 (15)	0.1839 (2)	0.0607 (8)	
O8	0.2672 (3)	0.42416 (15)	0.07515 (19)	0.0626 (8)	
O9	0.4602 (2)	0.35127 (15)	0.13931 (19)	0.0591 (8)	
O10	0.3744 (3)	0.29628 (15)	0.2560 (2)	0.0620 (8)	
S1	0.37560 (12)	0.73425 (7)	0.42525 (10)	0.0830 (4)	
S2	0.92911 (16)	0.58114 (9)	0.2533 (2)	0.1581 (12)	
S3	0.5997 (2)	0.80848 (15)	0.03857 (12)	0.1449 (9)	
S4	0.76171 (14)	0.94434 (7)	0.44060 (10)	0.0824 (4)	

### Atomic displacement parameters (Å^2^)

**Table:**

	*U*^11^	*U*^22^	*U*^33^	*U*^12^	*U*^13^	*U*^23^
C1	0.104 (5)	0.101 (5)	0.051 (3)	−0.056 (4)	0.037 (3)	−0.032 (3)
C2	0.157 (7)	0.152 (7)	0.087 (5)	−0.096 (6)	0.049 (5)	−0.059 (5)
C3	0.136 (8)	0.183 (10)	0.086 (6)	−0.091 (7)	0.061 (6)	−0.050 (6)
C4	0.109 (8)	0.279 (16)	0.149 (11)	−0.091 (9)	0.071 (7)	−0.093 (10)
C5	0.069 (4)	0.206 (9)	0.136 (7)	−0.011 (5)	0.033 (5)	−0.087 (7)
C6	0.055 (3)	0.106 (5)	0.103 (5)	−0.006 (3)	0.022 (3)	−0.040 (4)
C7	0.075 (4)	0.062 (4)	0.182 (7)	0.027 (3)	−0.003 (4)	−0.045 (4)
C8	0.109 (5)	0.058 (4)	0.157 (7)	0.010 (3)	−0.028 (4)	0.001 (4)
C9	0.148 (5)	0.093 (5)	0.089 (5)	−0.048 (4)	0.014 (4)	0.035 (4)
C10	0.132 (5)	0.124 (6)	0.090 (5)	−0.064 (5)	0.025 (4)	0.009 (4)
C11	0.043 (3)	0.149 (5)	0.102 (5)	−0.028 (3)	0.019 (3)	−0.033 (4)
C12	0.058 (3)	0.143 (5)	0.119 (6)	−0.003 (4)	0.017 (4)	−0.036 (5)
C13	0.089 (4)	0.101 (4)	0.073 (4)	0.011 (3)	−0.029 (3)	−0.016 (3)
C14	0.112 (4)	0.090 (4)	0.072 (4)	0.012 (4)	−0.014 (3)	0.002 (3)
C15	0.068 (3)	0.042 (2)	0.059 (3)	−0.010 (2)	0.000 (2)	0.006 (2)
C16	0.093 (4)	0.053 (3)	0.088 (4)	−0.004 (3)	−0.015 (3)	0.018 (3)
C17	0.125 (6)	0.084 (4)	0.082 (5)	−0.026 (4)	−0.021 (4)	0.046 (4)
C18	0.131 (6)	0.102 (5)	0.065 (4)	−0.042 (5)	0.004 (4)	0.026 (4)
C19	0.094 (4)	0.077 (4)	0.064 (3)	−0.028 (3)	0.017 (3)	0.008 (3)
C20	0.063 (3)	0.045 (2)	0.053 (3)	−0.016 (2)	0.005 (2)	0.003 (2)
C21	0.046 (2)	0.064 (3)	0.088 (3)	−0.006 (2)	0.024 (2)	−0.006 (2)
C22	0.037 (2)	0.070 (3)	0.096 (3)	−0.005 (2)	0.006 (2)	0.004 (3)
C23	0.059 (3)	0.062 (3)	0.100 (4)	0.007 (2)	−0.008 (3)	0.032 (3)
C24	0.062 (3)	0.075 (3)	0.071 (3)	−0.005 (2)	−0.015 (2)	0.026 (2)
C25	0.086 (3)	0.080 (4)	0.050 (3)	−0.015 (3)	0.012 (3)	0.001 (3)
C26	0.075 (3)	0.077 (3)	0.073 (4)	−0.016 (3)	0.030 (3)	−0.008 (3)
C27	0.061 (3)	0.063 (3)	0.079 (3)	0.006 (2)	0.014 (3)	−0.022 (2)
C28	0.065 (3)	0.053 (3)	0.087 (3)	0.016 (2)	0.011 (3)	−0.007 (3)
C29	0.060 (3)	0.048 (3)	0.109 (5)	−0.005 (2)	0.004 (3)	−0.009 (3)
C30	0.050 (3)	0.059 (3)	0.164 (7)	−0.014 (3)	0.025 (3)	−0.012 (4)
C31	0.071 (4)	0.127 (6)	0.091 (5)	−0.001 (4)	0.017 (4)	−0.060 (5)
C32	0.052 (3)	0.059 (3)	0.055 (3)	0.001 (2)	0.011 (2)	0.011 (2)
Ba1	0.03838 (13)	0.03676 (13)	0.05013 (15)	−0.00420 (10)	0.00463 (10)	−0.00217 (11)
Co1	0.0583 (4)	0.0668 (4)	0.1027 (6)	−0.0035 (3)	0.0174 (4)	−0.0230 (4)
N1	0.069 (3)	0.086 (4)	0.149 (5)	−0.022 (3)	0.037 (3)	−0.038 (3)
N2	0.065 (3)	0.065 (3)	0.146 (5)	−0.005 (2)	0.026 (3)	−0.016 (3)
N3	0.094 (4)	0.133 (5)	0.103 (4)	0.026 (3)	0.003 (4)	−0.045 (4)
N4	0.075 (3)	0.058 (3)	0.076 (3)	−0.012 (2)	0.021 (2)	−0.005 (2)
O1	0.088 (3)	0.093 (3)	0.082 (3)	0.039 (2)	0.015 (2)	−0.012 (2)
O2	0.110 (3)	0.047 (2)	0.099 (3)	−0.014 (2)	−0.032 (2)	0.011 (2)
O3	0.084 (3)	0.093 (3)	0.102 (3)	−0.038 (2)	0.033 (2)	−0.014 (2)
O4	0.052 (2)	0.118 (3)	0.093 (3)	0.014 (2)	0.001 (2)	−0.026 (2)
O5	0.092 (3)	0.081 (2)	0.055 (2)	0.020 (2)	0.0056 (19)	0.0089 (18)
O6	0.0497 (17)	0.0530 (17)	0.070 (2)	0.0003 (14)	0.0181 (15)	0.0139 (15)
O7	0.0429 (16)	0.0508 (17)	0.084 (2)	−0.0001 (13)	0.0014 (16)	0.0086 (16)
O8	0.0623 (19)	0.065 (2)	0.0551 (19)	−0.0068 (16)	−0.0013 (15)	0.0067 (16)
O9	0.0550 (18)	0.0599 (19)	0.063 (2)	−0.0028 (15)	0.0122 (15)	−0.0126 (16)
O10	0.0638 (19)	0.0465 (17)	0.078 (2)	0.0162 (14)	0.0207 (17)	0.0085 (16)
S1	0.0646 (8)	0.0724 (9)	0.1137 (13)	0.0111 (7)	0.0210 (8)	0.0001 (8)
S2	0.0730 (11)	0.0722 (11)	0.345 (4)	−0.0107 (8)	0.0797 (17)	−0.0492 (16)
S3	0.1433 (19)	0.208 (3)	0.0861 (14)	−0.0415 (18)	0.0277 (13)	−0.0441 (15)
S4	0.0922 (10)	0.0656 (9)	0.0870 (10)	0.0076 (7)	0.0114 (8)	−0.0161 (7)

### Geometric parameters (Å, º)

**Table:**

C1—C6	1.383 (9)	C20—O6	1.386 (5)
C1—C2	1.393 (9)	C21—O6	1.439 (5)
C1—O5	1.409 (7)	C21—C22	1.482 (7)
C2—C3	1.424 (12)	C21—H21A	0.9700
C2—H2	0.9300	C21—H21B	0.9700
C3—C4	1.392 (14)	C22—O7	1.424 (5)
C3—H3	0.9300	C22—H22A	0.9700
C4—C5	1.356 (13)	C22—H22B	0.9700
C4—H4	0.9300	C23—O7	1.443 (6)
C5—C6	1.386 (9)	C23—C24	1.487 (8)
C5—H5	0.9300	C23—H23A	0.9700
C6—O1	1.325 (8)	C23—H23B	0.9700
C7—O1	1.476 (7)	C24—O8	1.421 (6)
C7—C8	1.512 (10)	C24—H24A	0.9700
C7—H7A	0.9700	C24—H24B	0.9700
C7—H7B	0.9700	C25—O8	1.426 (6)
C8—O2	1.350 (8)	C25—C26	1.500 (7)
C8—H8A	0.9700	C25—H25A	0.9700
C8—H8B	0.9700	C25—H25B	0.9700
C9—C10	1.474 (10)	C26—O9	1.423 (6)
C9—O2	1.525 (8)	C26—H26A	0.9700
C9—H9A	0.9700	C26—H26B	0.9700
C9—H9B	0.9700	C27—O9	1.431 (6)
C10—O3	1.368 (7)	C27—C28	1.464 (7)
C10—H10A	0.9700	C27—H27A	0.9700
C10—H10B	0.9700	C27—H27B	0.9700
C11—O3	1.442 (7)	C28—O10	1.428 (5)
C11—C12	1.468 (10)	C28—H28A	0.9700
C11—H11A	0.9700	C28—H28B	0.9700
C11—H11B	0.9700	C29—N1	1.142 (7)
C12—O4	1.397 (7)	C29—S1	1.606 (6)
C12—H12A	0.9700	C30—N2	1.138 (7)
C12—H12B	0.9700	C30—S2	1.609 (7)
C13—O4	1.422 (7)	C31—N3	1.142 (9)
C13—C14	1.428 (9)	C31—S3	1.600 (9)
C13—H13A	0.9700	C32—N4	1.153 (6)
C13—H13B	0.9700	C32—S4	1.603 (5)
C14—O5	1.386 (6)	Ba1—O3	2.769 (4)
C14—H14A	0.9700	Ba1—O9	2.776 (3)
C14—H14B	0.9700	Ba1—O2	2.776 (3)
C15—O10	1.376 (5)	Ba1—O6	2.803 (3)
C15—C20	1.379 (7)	Ba1—O10	2.806 (3)
C15—C16	1.392 (6)	Ba1—O8	2.808 (3)
C16—C17	1.388 (9)	Ba1—O5	2.821 (3)
C16—H16	0.9300	Ba1—O7	2.824 (3)
C17—C18	1.351 (10)	Ba1—O4	2.832 (3)
C17—H17	0.9300	Ba1—O1	2.839 (4)
C18—C19	1.390 (8)	Co1—N3	1.932 (7)
C18—H18	0.9300	Co1—N2	1.947 (5)
C19—C20	1.371 (7)	Co1—N4	1.950 (4)
C19—H19	0.9300	Co1—N1	1.953 (5)
			
C6—C1—C2	123.4 (7)	O8—C25—H25B	110.1
C6—C1—O5	113.5 (5)	C26—C25—H25B	110.1
C2—C1—O5	123.1 (8)	H25A—C25—H25B	108.4
C1—C2—C3	117.0 (9)	O9—C26—C25	112.2 (4)
C1—C2—H2	121.5	O9—C26—H26A	109.2
C3—C2—H2	121.5	C25—C26—H26A	109.2
C4—C3—C2	117.5 (9)	O9—C26—H26B	109.2
C4—C3—H3	121.2	C25—C26—H26B	109.2
C2—C3—H3	121.2	H26A—C26—H26B	107.9
C5—C4—C3	124.7 (11)	O9—C27—C28	109.9 (4)
C5—C4—H4	117.7	O9—C27—H27A	109.7
C3—C4—H4	117.7	C28—C27—H27A	109.7
C4—C5—C6	118.2 (11)	O9—C27—H27B	109.7
C4—C5—H5	120.9	C28—C27—H27B	109.7
C6—C5—H5	120.9	H27A—C27—H27B	108.2
O1—C6—C1	115.7 (5)	O10—C28—C27	106.8 (4)
O1—C6—C5	125.1 (8)	O10—C28—H28A	110.4
C1—C6—C5	119.1 (8)	C27—C28—H28A	110.4
O1—C7—C8	106.3 (5)	O10—C28—H28B	110.4
O1—C7—H7A	110.5	C27—C28—H28B	110.4
C8—C7—H7A	110.5	H28A—C28—H28B	108.6
O1—C7—H7B	110.5	N1—C29—S1	178.6 (6)
C8—C7—H7B	110.5	N2—C30—S2	179.5 (7)
H7A—C7—H7B	108.7	N3—C31—S3	177.3 (7)
O2—C8—C7	107.3 (6)	N4—C32—S4	178.8 (5)
O2—C8—H8A	110.3	O3—Ba1—O9	77.29 (11)
C7—C8—H8A	110.3	O3—Ba1—O2	60.35 (14)
O2—C8—H8B	110.3	O9—Ba1—O2	116.84 (13)
C7—C8—H8B	110.3	O3—Ba1—O6	167.84 (11)
H8A—C8—H8B	108.5	O9—Ba1—O6	102.94 (9)
C10—C9—O2	112.3 (5)	O2—Ba1—O6	128.00 (12)
C10—C9—H9A	109.2	O3—Ba1—O10	119.38 (11)
O2—C9—H9A	109.2	O9—Ba1—O10	57.53 (9)
C10—C9—H9B	109.2	O2—Ba1—O10	173.07 (11)
O2—C9—H9B	109.2	O6—Ba1—O10	53.62 (9)
H9A—C9—H9B	107.9	O3—Ba1—O8	97.11 (11)
O3—C10—C9	108.3 (5)	O9—Ba1—O8	60.15 (10)
O3—C10—H10A	110.0	O2—Ba1—O8	80.01 (11)
C9—C10—H10A	110.0	O6—Ba1—O8	93.41 (9)
O3—C10—H10B	110.0	O10—Ba1—O8	93.27 (9)
C9—C10—H10B	110.0	O3—Ba1—O5	95.21 (12)
H10A—C10—H10B	108.4	O9—Ba1—O5	125.10 (10)
O3—C11—C12	110.1 (5)	O2—Ba1—O5	104.54 (12)
O3—C11—H11A	109.6	O6—Ba1—O5	74.58 (11)
C12—C11—H11A	109.6	O10—Ba1—O5	82.38 (10)
O3—C11—H11B	109.6	O8—Ba1—O5	167.53 (11)
C12—C11—H11B	109.6	O3—Ba1—O7	134.32 (12)
H11A—C11—H11B	108.2	O9—Ba1—O7	114.23 (9)
O4—C12—C11	113.8 (6)	O2—Ba1—O7	76.15 (11)
O4—C12—H12A	108.8	O6—Ba1—O7	56.94 (9)
C11—C12—H12A	108.8	O10—Ba1—O7	102.06 (9)
O4—C12—H12B	108.8	O8—Ba1—O7	60.04 (10)
C11—C12—H12B	108.8	O5—Ba1—O7	109.30 (11)
H12A—C12—H12B	107.7	O3—Ba1—O4	60.39 (14)
O4—C13—C14	111.6 (5)	O9—Ba1—O4	75.46 (10)
O4—C13—H13A	109.3	O2—Ba1—O4	113.47 (12)
C14—C13—H13A	109.3	O6—Ba1—O4	107.70 (11)
O4—C13—H13B	109.3	O10—Ba1—O4	70.08 (11)
C14—C13—H13B	109.3	O8—Ba1—O4	134.20 (11)
H13A—C13—H13B	108.0	O5—Ba1—O4	55.06 (11)
O5—C14—C13	107.3 (5)	O7—Ba1—O4	162.51 (11)
O5—C14—H14A	110.3	O3—Ba1—O1	92.27 (13)
C13—C14—H14A	110.3	O9—Ba1—O1	169.33 (11)
O5—C14—H14B	110.3	O2—Ba1—O1	58.08 (14)
C13—C14—H14B	110.3	O6—Ba1—O1	86.79 (12)
H14A—C14—H14B	108.5	O10—Ba1—O1	128.21 (12)
O10—C15—C20	114.5 (4)	O8—Ba1—O1	124.38 (10)
O10—C15—C16	125.1 (5)	O5—Ba1—O1	52.89 (11)
C20—C15—C16	120.4 (5)	O7—Ba1—O1	74.65 (10)
C17—C16—C15	118.1 (6)	O4—Ba1—O1	97.59 (11)
C17—C16—H16	120.9	N3—Co1—N2	103.8 (2)
C15—C16—H16	120.9	N3—Co1—N4	108.5 (2)
C18—C17—C16	121.2 (6)	N2—Co1—N4	116.05 (19)
C18—C17—H17	119.4	N3—Co1—N1	111.0 (3)
C16—C17—H17	119.4	N2—Co1—N1	115.1 (2)
C17—C18—C19	120.7 (6)	N4—Co1—N1	102.42 (19)
C17—C18—H18	119.6	C29—N1—Co1	162.1 (5)
C19—C18—H18	119.6	C30—N2—Co1	162.5 (6)
C20—C19—C18	118.9 (6)	C31—N3—Co1	172.9 (7)
C20—C19—H19	120.5	C32—N4—Co1	163.0 (4)
C18—C19—H19	120.5	C6—O1—C7	121.8 (5)
C19—C20—C15	120.6 (5)	C6—O1—Ba1	114.9 (4)
C19—C20—O6	124.7 (5)	C7—O1—Ba1	113.2 (4)
C15—C20—O6	114.8 (4)	C8—O2—C9	110.6 (5)
O6—C21—C22	106.2 (4)	C8—O2—Ba1	122.1 (4)
O6—C21—H21A	110.5	C9—O2—Ba1	108.0 (4)
C22—C21—H21A	110.5	C10—O3—C11	111.4 (5)
O6—C21—H21B	110.5	C10—O3—Ba1	123.1 (4)
C22—C21—H21B	110.5	C11—O3—Ba1	119.3 (4)
H21A—C21—H21B	108.7	C12—O4—C13	115.6 (5)
O7—C22—C21	108.8 (4)	C12—O4—Ba1	110.3 (4)
O7—C22—H22A	109.9	C13—O4—Ba1	121.7 (3)
C21—C22—H22A	109.9	C14—O5—C1	119.9 (5)
O7—C22—H22B	109.9	C14—O5—Ba1	123.0 (4)
C21—C22—H22B	109.9	C1—O5—Ba1	113.7 (3)
H22A—C22—H22B	108.3	C20—O6—C21	119.9 (4)
O7—C23—C24	112.1 (4)	C20—O6—Ba1	115.5 (2)
O7—C23—H23A	109.2	C21—O6—Ba1	118.8 (2)
C24—C23—H23A	109.2	C22—O7—C23	112.5 (4)
O7—C23—H23B	109.2	C22—O7—Ba1	121.0 (3)
C24—C23—H23B	109.2	C23—O7—Ba1	110.3 (3)
H23A—C23—H23B	107.9	C24—O8—C25	112.4 (4)
O8—C24—C23	108.0 (4)	C24—O8—Ba1	119.8 (3)
O8—C24—H24A	110.1	C25—O8—Ba1	119.4 (3)
C23—C24—H24A	110.1	C26—O9—C27	114.5 (4)
O8—C24—H24B	110.1	C26—O9—Ba1	111.1 (3)
C23—C24—H24B	110.1	C27—O9—Ba1	121.3 (3)
H24A—C24—H24B	108.4	C15—O10—C28	119.9 (4)
O8—C25—C26	108.0 (4)	C15—O10—Ba1	115.6 (3)
O8—C25—H25A	110.1	C28—O10—Ba1	117.2 (3)
C26—C25—H25A	110.1		
